# Feasibility and safety of rapid glofitamab ramp-up

**DOI:** 10.1038/s41375-026-03003-3

**Published:** 2026-07-01

**Authors:** Celine H. Liem, Rebecca Wurm-Kuczera, David Böckle, Marie Anne-Catherine Neumann, Marcel Teichert, Urban Novak, Merle Klein Helmkamp, Evgenii Shumilov, Nina Malburg, Tim Versteegen, Fabian Görke, Giulia Magno, Susanne Ghandili, Joseph Kauer, Konstantinos Christofyllakis, Thomas Melchardt, Peter Dreger, Francis Ayuk, Marion Subklewe, Vladan Vučinić, Christiane Pott, Hans Christian Reinhardt, Georg Lenz, Bastian von Tresckow, Thomas Pabst, Peter Borchmann, Antonia Busse, Ulrich Keller, Björn Chapuy

**Affiliations:** 1 Department of Hematology, Oncology, and Cancer Immunology, Campus Benjamin Franklin, Charité-Universitätsmedizin Berlin, Corporate Member of Freie Universität Berlin and Humboldt-Universität zu Berlin, Berlin, Germany; 2 German Lymphoma Alliance (GLA), Alliance, Germany; 3 Department I of Internal Medicine, Center for Integrated Oncology Aachen Bonn Cologne Duesseldorf (CIO ABCD), Faculty of Medicine and University Hospital of Cologne, University of Cologne, Cologne, Germany; 4 Department of Hematology and Stem Cell Transplantation, West German Cancer Center, University Hospital Essen, University of Duisburg-Essen, Essen, Germany; 5 Department of Medical Oncology, Inselspital, Bern University Hospital, University of Bern, Bern, Switzerland; 6 Department of Medicine A, Hematology, Oncology and Pneumology, University Hospital Muenster, Münster, Germany; 7 Second Medical Department, University Hospital Schleswig-Holstein, Kiel, Germany; 8 Department of Hematology, Cell Therapy, Hemostaseology and Infectious Diseases, University Hospital Leipzig, Leipzig, Germany; 9 Department of Medicine III, University Hospital, Ludwig-Maximilians-University Munich, Munich, Germany; 10 German Cancer Consortium, Partner Site Munich, Ludwig-Maximilians-University Munich, Munich, Germany; 11 Department of Oncology, Hematology, and Bone Marrow Transplantation with Section Pneumology, University Medical Center Hamburg-Eppendorf, Hamburg, Germany; 12 Internal Medicine V, Hematology, Oncology and Rheumatology, Heidelberg University Hospital, Heidelberg, Germany; 13 Department of Internal Medicine 1 (Oncology, Hematology, Clinical Immunology, and Rheumatology), Saarland University Medical School, Homburg/Saar, Germany; 14 Department of Internal Medicine III with Haematology, Medical Oncology, Haemostaseology, Infectiology and Rheumatology, Cancer Center, Paracelsus Medical University Salzburg, Salzburg, Austria; 15 Department of Stem Cell Transplantation, University Medical Center Hamburg-Eppendorf, Hamburg, Germany; 16 Fraunhofer Institute for Cell Therapy and Immunology, Leipzig, Germany; 17 German Cancer Consortium, Partner site Essen/Düsseldorf, University Hospital Essen, Essen, Germany; 18 Center for Molecular Biotechnology, University Hospital Essen, Essen, Germany; 19 German Cancer Consortium, Partner Site Berlin, a Partnership between German Cancer Consortium and Charité-Universitätsmedizin Berlin, Berlin, Germany; 20 Max Delbrück Center for Molecular Medicine in the Helmholtz Association, Berlin, Germany; 21 Cluster of Excellence ImmunoPreCept, Charité-Universitätsmedizin Berlin, Berlin, Germany

**Keywords:** Cancer immunotherapy, Immunotherapy, B-cell lymphoma

## Introduction

Diffuse large B-cell lymphoma (DLBCL) is clinically and molecularly a heterogeneous disease with recognized transcriptional (germinal center B-cell-type, GCB; activated B-cell-type, ABC) and genetic subtypes (DLB*class*, LymphGen) [1–7]. State-of-the-art immunochemotherapy cures approximately 75% of patients [8]. Refractory or relapsed disease (r/r LBCL) is often characterized by rapid lymphoma proliferation and clinical need for fast-acting treatments. Prognosis in r/r LBCL is substantially diminished despite the use of engineered autologous T-cells with a CD19-targeting chimeric antigen receptor (CAR-T) [9]. Notably, approximately 50% of patients relapse after or are refractory to CD19-directed CAR-T-cell therapy with different determinants of resistant disease, highlighting the medical need for treatment optimization following CAR-T [10, 11].

Glofitamab is a CD20xCD3 bispecific, T-cell-engaging monoclonal antibody that mediates direct recruitment and activation of autologous T-cells in the vicinity of lymphoma cells [12]. Following secretion of granzymes and perforins, lymphoma cells are eventually killed. In the pivotal trial, single-agent glofitamab achieved a complete response (CMR) rate of 39% (95% confidence interval [95% CI], 32–48%), which translated into a 12-month progression-free survival (PFS) rate of 37% (95% CI, 28–46%) [12]. This data led to the approval of glofitamab for patients with relapsed or refractory DLBCL not otherwise specified, and LBCL arising from follicular lymphoma, after two or more lines of systemic therapy [12]. Several studies have also confirmed glofitamab’s safety and efficacy in real-world analyses (RWA) [13, 14].

To mitigate cytokine release syndrome (CRS) and immune effector cell–associated neurotoxicity syndrome (ICANS), the standard glofitamab protocol requires pretreatment with obinutuzumab and a 3-week step-up dosing of glofitamab until reaching the full therapeutic dose. The potentially delayed disease control during the ramp-up phase of glofitamab is contrasted by high proliferation and often high tumor burden at diagnosis of r/r LBCL, limiting the use of single-agent glofitamab and resulting in the development of the BiCAR trial [15] and other off-label accelerated step-up protocols in clinical practice. To assess whether the full dose of glofitamab can be administered more rapidly without compromising patient safety and thereby accelerating disease control in real-world settings, we conducted a retrospective, multicenter study enrolling patients who had received accelerated glofitamab.

## Subjects and methods

In this retrospective study, we analyzed 83 patients with r/r LBCL from 11 centers across Germany, Austria, and Switzerland between 12/2020 and 11/2025. Patients received glofitamab with an accelerated step-up dosing ranging from 4 to 14 days. The decision to apply accelerated step-up dosing and the timing between individual dose steps were based on physician judgment in the real-world setting. Key factors influencing these decisions included disease kinetics, tumor burden, urgency of disease control, patient performance status, and tolerability of preceding doses. Patients in the accelerated group were either retrospectively assigned to the rapid glofitamab group (“Rapid Glo”) when the full step-up dosing was administered within 1 week or to the BiCAR-like group (“BiCAR-like”) when step-up dosing occurred within 1–2 weeks [15]. Clinical data were derived from electronic medical records and pseudonymized at the respective clinical center before analysis. The study was approved by the IRB of Charité-Universitätsmedizin Berlin (EA1/211/25). CRS and ICANS were evaluated according to the ASTCT Consensus Grading. Adverse events (AE) were categorized using the Common Terminology Criteria for Adverse Events (CTCAE V5) from the 2.5 mg glofitamab dose until the second 30 mg dose of glofitamab. Standard glofitamab AEs were obtained from the pivotal trial or our real-world analysis [12, 13]. Supportive therapy, response, and clinical outcome assessments are detailed in the Supplemental Appendix.

## Results

### Accelerated glofitamab regimens

**Fig. 1:**
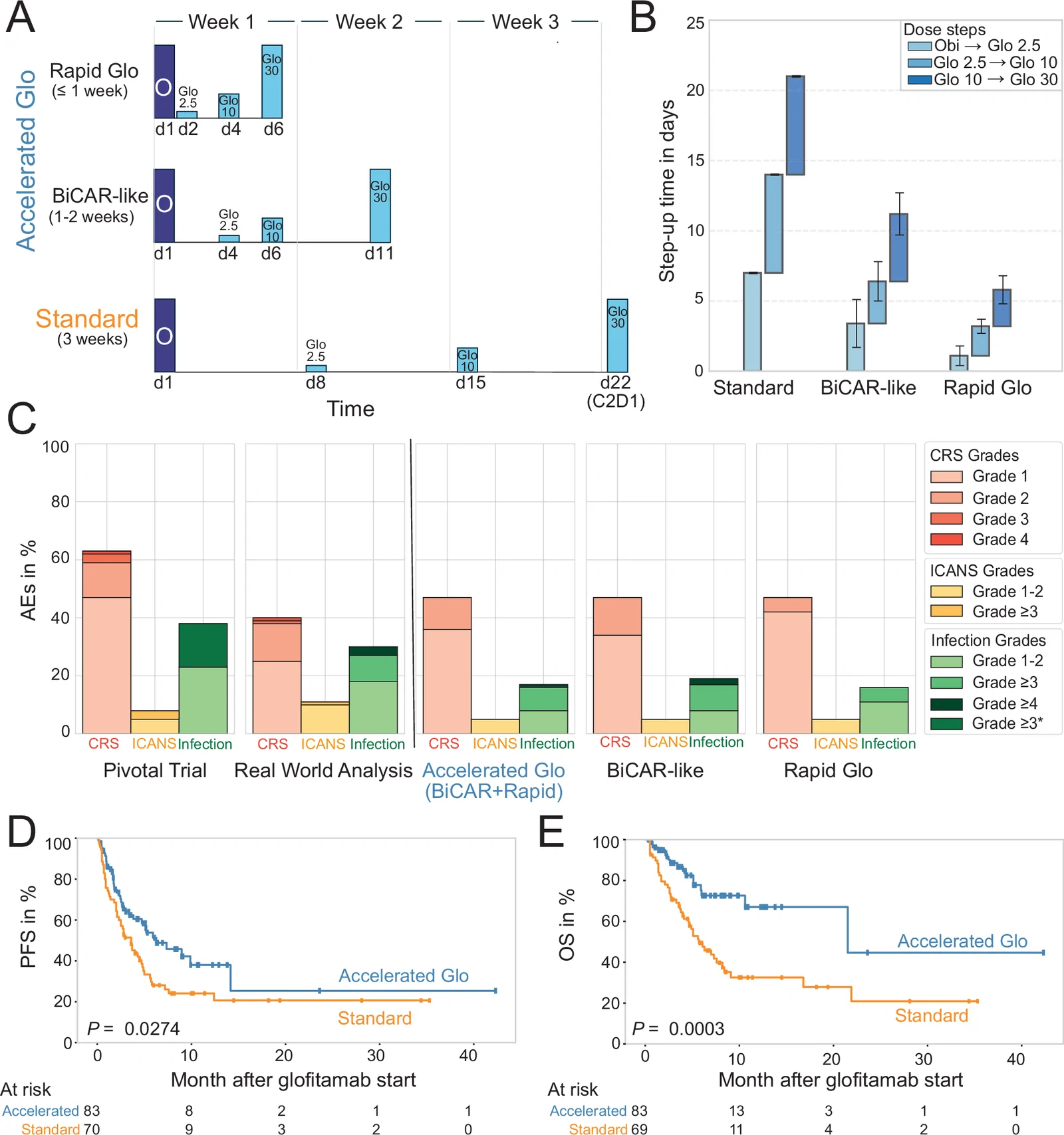
Accelerated glofitamab dosing schedules and their reported adverse events and outcomes. **A** Comparison of standard dosing with the accelerated dosing schedules. “Rapid Glo” ( ≤ 1 week), top panel, “BiCAR-like” (1-2 weeks), middle panel; standard dosing (3 weeks), lower panel. Obinutuzumab (O), purple box on d1; glofitamab step-up doses, scaled blue boxes (Glo 2.5, 2.5 mg; Glo 10, 10 mg; Glo 30, 30 mg). Time on the x-axis in days. Days of treatment are indicated below the boxes. **B** Timing between each step per group is shown on the right. Comparison of dosing schedule between standard dosing, “BiCAR-like”, and “Rapid Glo” group. Boxes represent the mean days between glofitamab dose steps as indicated, whiskers indicate the standard deviation in days. **C** CRS, ICANS, and infection rate. Comparison of standard dosing (data derived from the pivotal trial in *Dickinson* et al.* NEJM 2022*). Infections are graded in three categories (grade 1-2, grade 3, and grade ≥ 4); * for Dickinson et al., infections are graded in 2 categories (grade 1-2, and grade ≥ 3). Percentage indicates AE per patient with CRS/ICANS/infection (all grades). **D**, **E** PFS (**D**) and OS (**E**) for all patients treated with accelerated dosing (“BiCAR-like” and “Rapid Glo”, blue curve) and standard dosing (from the Real-World Analysis *in Shumilov, Wurm-Kuczera, Kerkhoff et al. Blood Adv. 2024*, orange curve) on the left. The *P*-value was obtained by a log-rank test.

The study enrolled 83 patients with r/r LBCL who received an accelerated glofitamab dosing at the treating physician’s discretion and compared safety and efficacy to 70 real-world patients with r/r LBCL [13] who received glofitamab using the standard 3-week ramp-up (“Standard”, Fig. 1A). All patients received 1000 mg obinutuzumab once before the first dose of glofitamab. Glofitamab was administered in the dose steps of 2.5 mg, 10 mg, and 30 mg; after reaching the full dose of 30 mg, glofitamab was administered every 21 days. All 83 enrolled patients had received the glofitamab step-up in a shorter timespan than the standard 3-week step-up. The median time between the administration of obinutuzumab and the first full dose of 30 mg glofitamab was 11 days (range: 4–14). The majority (77% [64/83]) of patients had received all doses similar to the 11-day dosing schedule used in the BiCAR-trial [15] (“BiCAR-like”, Fig. 1A/B), while the remainder of 23% (19/83) had received the step-up doses in less than a week with a median of 6 days (range: 4–7) from obinutuzumab until the first full dose of glofitamab (“Rapid Glo”, Fig. 1A/B). At the time of analysis, 19 patients (23%) had still ongoing glofitamab treatment.

### Patient characteristics

The median age at diagnosis was 63 years (range: 28–81), and patients had a slight male predominance (Table 1). The most common histological subtype was DLBCL-NOS (89%[74/83]), followed by High Grade B-Cell Lymphoma (HGBCL) with *MYC* and *BCL2* translocation (11%[9/83]) (Table 1). Twenty-four percent of patients had an ECOG of 2 or higher, the median lactate dehydrogenase (LDH) was 291 U/l (range:158–6778), 74% (61/83) had advanced-staged disease, and 69% (57/83) had extranodal manifestations, resulting in 44% of patients presenting with an intermediate or high clinical risk according to the international prognostic index (IPI) (Table 1). The median number of prior treatment lines was 3 (range:1–7), with 48% (40 patients) of patients having failed CAR-T-cell therapy before glofitamab; more than a third received CAR-T-cell therapy after treatment with glofitamab (35% [29/83])(Table 1). Most patients received glofitamab as monotherapy (87% [72/83]; Table S1). Overall, clinical baseline characteristics were generally comparable between registration trial, real-world analyses, and the two accelerated glofitamab regimens (“BiCAR-like” and “Rapid Glo”) (Table 1).

**Table 1:** Patient characteristics.

Parameters	Accelerated Glo (“BiCAR-like”+ “Rapid Glo” (≤2 weeks)	BiCAR-like (1-2 weeks)	Rapid Glo (≤1 week)	Standard dosing (Pivotal trial^a^)	Standard Dosing (RWA^b^)
**No. of patients**	83 (100%)	64 (77%)	19 (23%)	155	70
**Age (years)**	63 (28–81)	62 (28–81)	63 (32–81)	66 (21–90)	62 (23–94)
**Male**	48 (58%)	36 (56%)	12 (63%)	100 (65%)	41 (59%)
**Female**	35 (42%)	28 (44%)	7 (37%)	55 (35%)	29 (41%)
**DLBCL subtypes:**					
DLBCL NOS	74 (89%)	56 (88%)	18 (95%)	110 (71%)	40 (57%)
DLBCL GCB	38 (46%)	27 (42%)	11 (58%)	na	13 (19%)
DLBCL non-GCB	21 (25%)	19 (30%)	2 (11%)	na	16 (23%)
GCB or non-GCB unknown	15 (18%)	10 (16%)	5 (26%)	na	11 (16%)
HGBCL (*MYC/BCL2*-translocation)	9 (11%)	8 (12%)	1 (5%)	11 (7%)	9 (13%)
**No. of treatments before glo**	3 (1–7)	3 (1–5)	2 (1–7)	3 (2–7)	4 (2–14)
Prior CAR-T-cell therapy	40 (48%)	32 (50%)	8 (42%)	na	50 (71%)
Prior autoPBSC	9 (11%)	7 (11%)	2 (11%)	na	22 (31%)
**Median dose step-up time in days (range)** (Obi to full dose glo)	11 (4–14)	11 (8–14)	6 (4–7)	21	21
**Median time in days (range)**					
Obi to 2,5 mg glofitamab	3 (0–8)	3 (0–8)	1 (0–3)	7	7
2,5 mg to 10 mg glofitamab	2 (0–6)	3 (0–6)	2 (1–3)	7	7
10 to 30 mg glofitamab	5 (2–9)	5 (2–9)	2 (2–5)	7	7
**Median no. of cycles**	3 (1–14)	2 (1–14)	3 (1–12)	5 (1–13)	4 (1–12)
**Ongoing glofitamab treatment**	19 (23%)	13 (20%)	6 (32%)	na	9 (13%)
**Treatment after glofitamab:**					
CAR-T-cell therapy	30 (35%)	24 (38%)	6 (32%)	na	3 (12%)
AlloPBSC	3 (4%)	2 (3%)	1 (5%)	na	3 (12%)
Loncastuximab	9 (11%)	5 (8%)	4 (21%)	na	5 (20%)
Other (e.g. VIPOR-P, ADC, …)	14 (17%)	13 (20%)	1 (5%)	na	14 20%)
None	27 (33%)	20 (31%)	7 (37%)	na	35 (50%)
**Glofitamab monotherapy**	72 (87%)	56 (87%)	16 (84%)	155 (100%)	70 (100%)
**Glofitamab + chemotherapy**	9 (11%)	7 (11%)	2 (11%)	na	na
**Glofitamab + other agents**	2 (2%)	1 (2%)	1 (5%)	na	na
**LDH (U/l)**	291 (158–6778)	290.5 (158 –6778)	294 (161 –792)	>250	400 (157–1799)
**Bulky disease (>7,5 cm)**	15 (18%)	14 (22%)	1 (5%)	>6 cm: 64 (42%) >10 cm: 18 (12%)	22 (32%)
**Extranodal manifestations**	57 (69%)	44 (69%)	13 (68%)	95 (61%)	23 (36%)
**IPI**					
0-1	17 (20%)	13 (20%)	4 (21%)	na	8 (13%)
2	30 (36%)	22 (34%)	8 (42%)	na	9 (14%)
3	27 (33%)	21 (33%)	6 (32%)	na	26 (41%)
4-5	9 (11%)	8 (13%)	1 (5%)	na	20 (32%)
**Stage (Ann-Arbor)**					
I	5 (6%)	4 (6%)	1 (5%)	10 (6%)	na
II	16 (19%)	12 (19%)	4 (21%)	25 (16%)	na
III	13 (16%)	11 (17%)	2 (11%)	31 (20%)	na
IV	48 (58%)	36 (56%)	12 (63%)	85 (55%)	na
Unknown	1 (1%)	1 (2%)	0	3 (2%)	
**ECOG**					
0	26 (31%)	18 (28%)	8 (42%)	69 (45%)	grade ≤1: 60%
1	37 (45%)	32 (50%)	5 (26.3%)	84 (55%)	
2	15 (18%)	10 (15%)	5 (26.3%)	na	
3	4 (5%)	3 (5%)	1 (5.3%)	na	grade ≥2: 40%
4	1 (1%)	1 (2%)	0	na	

*HGBCL* High-grade B-cell-lymphoma, *BCL2/BCL6* B-cell lymphoma 2/6, *MYC* myelocytomatosis oncogene, *autoPBSC* autologous peripheral blood stem cell transplantation, *alloPBSC* allogeneic peripheral blood stem cell transplantation, *ADC* antibody-drug conjugate, *IPI* International Prognostic Index, *ECOG* Eastern Cooperative Oncology Group performance status score. *Obi* Obinutuzumab, *Glo* Glofitamab. *VIPOR-P* venetoclax, ibrutinib, prednisone, obinutuzumab, lenalidomide ± polatuzumab vedotin, *LDH* lactate dehydrogenase.

^a^Registration trial, *Dickinson* et al*.*NEJM [12].

^b^*Shumilov, Wurm-Kuczera, Kerkhoff* et al. Blood Advances [13].

### CRS, ICANS, and infections

CRS occurred in 47% (39/83) of patients; most CRS were grade 1 (36% [30/83]) and occurred after the first dose of 2.5 mg glofitamab (Fig. 1C and Table S2). CRS rates were similar in the “BiCAR-like” and “Rapid Glo” groups (Fig. 1C and Table S2) and remained consistent when stratified by treatment modality (glofitamab monotherapy vs. glofitamab/chemotherapy combination therapy; *p* = 0.33; Table S7). Only 24% of patients with CRS received CRS-specific treatment with steroids or tocilizumab (Table S3). Notably, no grade 3 or higher CRS events occurred. ICANS was rare (5% [4/83] and limited to low grades (grades 1–2); no ICANS grade 3 or higher was detected (Fig. 1C and Table S2). One patient experienced a grade 4 tumor lysis syndrome with acute kidney injury, who continued treatment after recovery (Table S2). Infections were reported in 18% (15/83) of patients, with 10% of patients experiencing grade 3–4 infections without a specific clustering of opportunistic pathogens (Tables S2 and S4). Overall, 7% (6/83) of patients were admitted to the intensive care unit, and all patients had a quick and full recovery (Tables S2 and S5).

### Response rates, time to response, and outcome

The best overall response rate for accelerated glofitamab was 53% (40/83; “Rapid Glo”, 47%; “BiCAR-like”, 55%), with 12% (10/83) of patients achieving CMRs/CRs (Table S6). With a median follow-up time of 5.1 months (“BiCAR-like”, 6.1 months; “Rapid Glo”, 4.2 months), the median time to response (TTR) was 1.2 months (range:0.3–8.8 months) (Table S6). The TTR was shorter with accelerated glofitamab protocols (accelerated, 1.2 months; standard, 1.8 months, *p* = 0.098), with TTR in “BiCAR-like” of 1.4 months and “Rapid Glo” of even 1.1 months (Table S6). These responses translated into a significantly increased median PFS of 6.3 months for the accelerated group compared to 3.6 months for the standard group (log-rank test, *P* = 0.027) (Fig. 1D and Table S6). Similarly, the median OS of 21.5 months for the accelerated group was significantly higher than the standard glofitamab group (mOS 5.7 months, *P* = 0.0003) (Fig. 1E and Table S6). At the time of analysis, 80% (66/83) of patients were still alive. No significant differences were found in the PFS between “Rapid Glo” and “BiCAR-like” group (log-rank *P* = 0.143) or for patients with and without prior CAR-T-cell therapy (log-rank *P* = 0.143).

## Discussion

This multicenter retrospective real-world study assessed the safety and efficacy of accelerated glofitamab step-up dosing in patients with r/r LBCL. The most common AEs were CRS (47%) and infections (18%). The CRS rate with accelerated protocols was lower than in the pivotal trial (63%) [12], similar to standard glofitamab ramp-up RWAs (27–40%) [13, 14], and higher compared to the recently reported BiCAR trial (13%) (Table S2) [15].

ICANS were similarly infrequent (5%) as reported in earlier studies [12–15]. Importantly, patients with accelerated step-up dosing experienced no grade 3 or higher CRS or ICANS. Accelerated glofitamab ramp-up resulted in a numerically lower rate of infections (18%) compared to the RWAs (30%, *P* = 0.09) [13, 14], and a significant reduction compared to the BiCAR trial (39%, *P* = 0.0116) [15] or the registration trial (38%, *P* = 0.002) [12]. The ICU admissions rate of 7% was also comparable to the RWA (10%)(Tbl. S5) [13, 14].

Patients with accelerated step-up dosing had a similar ORR (55%) and CR (12%) rate to previous studies with standard dosing (ORR: 46–55%; CR: 9–27%) [12, 13, 15], but efficacy comparisons need to be interpreted cautiously, given that they are based on non-matched external cohorts.

Notably, accelerated protocols sustained a longer median PFS of 6.3 months compared to the median PFS of prior studies (RWA, 3.6 months; BiCAR, 3.8 months; pivotal trial, 4.9 months) [12, 13, 15]. Likewise, accelerated glofitamab had a longer median OS of 21.5 months compared to the median OS of previously reported studies (RWA, 5.7 months; BiCAR, 14.7 months; pivotal trial, 11.5 months) [12, 13, 15]. Recognizing that many factors can contribute to the differences in outcome, particularly in cross-trial comparisons in the absence of matched pair analyses, this data support the conclusion that accelerated ramp-up protocols of glofitamab are not inferior to the reported outcome data of standard ramp-up.

Taken together, our study highlights that rapid administration of the glofitamab ( ≤ 1 week) is feasible without compromising patients’ safety or response, supporting its use in clinical practice for patients with r/r LBCL.

## Supplementary information

Supplemental Appendix

## Data Availability

The data that support the findings of this study are available in the supplementary material of this article and are available on request from the corresponding author.
