# Supplementary material for: Feasibility and safety of rapid glofitamab ramp-up
Source: Leukemia. 2026 Jul 1;40(9):2033–8. doi: 10.1038/s41375-026-03003-3 (PMC13506341; doi:10.1038/s41375-026-03003-3)
Supplement: Supplementary file 1 [file 41375_2026_3003_MOESM1_ESM.docx]

**Supplemental Appendix**

**to**

**Feasibility and Safety of Rapid Glofitamab Ramp-up**

by

Celine H Liem, Rebecca Wurm-Kuczera, David Böckle, Marie Anne-Catherine Neumann, Marcel Teichert, Urban Novak, Merle Klein Helmkamp, Evgenii Shumilov, Nina Malburg, Tim Versteegen, Fabian Görke, Giulia Magno, Susanne Ghandili, Joseph Kauer, Konstantinos Christofyllakis, Thomas Melchardt, Peter Dreger, Francis Ayuk, Marion Subklewe, Vladan Vučinić, Christiane Pott, Hans Christian Reinhardt, Georg Lenz, Bastian von Tresckow, Thomas Pabst, Peter Borchmann, Antonia Busse, Ulrich Keller, Björn Chapuy

**Contents**

***Supplemental Methods***…………………………………………………………….……………………2

Response and outcome assessment……………………………………………………………………2

Antimicrobial prophylaxis and growth factor support…………………………………………………..2

***Supplemental Tables***…………………………………………………………………………………….3

Supplemental Table 1: Co-administered therapies…………………………………………………….3

Supplemental Table 2: Comparison of AEs……………………………………………………………..4

Supplemental Table 3: CRS and ICANS Treatment……………………………………………………5

Supplemental Table 4: Grade ≥ 3 infections with accelerated Glo…………………………………...6

Supplemental Table 5: ICU admissions…………………………………………………………………7

Supplemental Table 6: Response parameters………………………………………………………….8

Supplemental Table 7: CRS rates stratified by treatment modality...………………….……………..9

**Supplemental Methods**

**Response and outcome assessment**

Response to treatment was assessed through computer tomography (CT) or positron emission tomography (PET)/CT-imaging and graded according to the Lugano response criteria (CR, complete response; CMR, complete metabolic response; PR, partial response; SD, stable disease; PD, progressive disease). Survival analyses were calculated from the first glofitamab infusion to the first documented disease progression (PFS) or death (OS) and using the Kaplan-Meier method. Time to follow up was estimated using the reverse Kaplan-Meier method.

**Antimicrobial prophylaxis and growth factor support**

All but 6 patients received anti-infective prophylactic therapy of cotrimoxazole and aciclovir or valaciclovir during treatment with glofitamab. Growth factors were administered according to local standards.

**Supplemental Tables**

**Supplemental Table 1: Co-administered therapies**

| **Therapies co-administered with glofitamab (n=11)** | **Accelerated Glo  (‘BiCAR-like’ + ’Rapid Glo’)** (≤ 2 weeks) | **BiCAR-like**  (1-2 weeks) | **Rapid Glo** (≤ 1 week) |
| --- | --- | --- | --- |
| **Chemotherapy** Gemcitabine and oxaliplatin R-ICE R-Methotrexate | 9 (10%) 7 (8%) 1 (1%) 1 (1%) | 7 (12%) 5 (8%) 1 (2%) 1 (2%) | 2 (10%) 2 (10%) 0 0 |
| **Other** Polatuzumab Intrathecal methotrexate | 2 (2%) 1 (1%) 1 (1%) | 1 (2%) 0 1 (2%) | 1 (5%) 1 (5%) 0 |

R: Rituximab; ICE: Ifosfamide, Carboplatin, Etoposide

**Supplemental Table 2: Comparison of AEs**

| **AE Parameter** | **Accelerated Glo (‘BiCAR-like’+ ’Rapid Glo’** (≤ 2 weeks) | **BiCAR-like**  (1-2 weeks) | **Rapid Glo** (≤ 1 week) | **Standard dosing**  (Pivotal trial^) | **Standard dosing** (RWA^#^) | **Standard dosing**  (RWA^$^) | **BiCAR trial*** |
| --- | --- | --- | --- | --- | --- | --- | --- |
| **No. of patients** | 83 | 64 | 19 | 155 | 70 | 89 Glofitamab* (156 Epcoritamab) | 46  (Cohort 1:  r/r DLBCL) |
| **CRS** None Grade 1 Grade 2 Grade ≥3 | 39 (47%) 44 (53%) 30 (36%) 9 (11%) 0 | 30 (47%) 34 (53%) 22 (34%) 8 (13%) 0 | 9 (47%) 10 (53%) 8 (42%) 1 (5%) 0 | 63% 37% 47% 12% 4% | 28 (40%) 42 (60%) 17 (25%) 9 (13%) 2 (2%) | 24 (27%) 65 (73%) na na 2 (2.4%) | 6 (13%) 40 (87%) 2 (4.3%) 4 (8.7%) 0 |
| **ICANS** None Grade 1-2 Grade ≥3 | 4 (5%) 79 (95%) 4 (5%) 0 | 3 (5%) 61 (95%) 3 (5%) 0 | 1 (5%) 18 (95%) 1 (5%) 0 | 8% 92% 5% 3% | 8 (11%) 62 (89%) 7 (10%) 1 (1%) | 6 (6.7%) 83 (93.3%) 5 (5.6%) 1 (1.1%) | 1 (2.2%) 45 98%) 1 (2.2%) 0 |
| **Infection** None Grade 1-2 Grade 3-4 | 15 (18%) 68 (82%) 7 (8%) 8 (10%) | 12 (19%) 52 (81%) 5 (8%) 7 (11%) | 3 (16%) 16 (84%) 2 (11%) 1 (5%) | 59 (38%) 96 (62%) 36 (23%) 23 (15%) | 21 (30%) 49 (70%) 13 (18%) 8 (12%) | death caused by infection:  4 (9.4%) | 18 (39%) 28 (61%) na na |
| **ICU admissions** | 6 (7%) | 5 (8%) | 1 (5%) | na | 7 (10%) | na | na |
| **Tumor lysis** | 1 (1%) | 1 (2%) | 0 | 1% | 2 (3%) | na | na |
| **AKI** | 3 (4%) | 1 (2%) | 2 (11%) | na | na | na | na |

AE: Adverse event; CRS: cytokine release syndrome; ICANS: immune effector cell-associated neurotoxicity syndrome. ICU: intensive care unit, AKI: Acute Kidney Injury. AE defined according to CTCAE (common terminology criteria for adverse events). *Data shown here: only the glofitamab-treated group. ^*Dickinson et al. NEJM 2022; # Shumilov, Wurm-Kuczera, Kerkhoff et al. Blood Advances 2024; $ Brooks et al. Blood 2025; * Cartron et al. Nature Cancer 2025.*

**Supplemental Table 3: CRS and ICANS Treatment**

| **Treatment** | **Accelerated Glo (‘BiCAR-like’+ ’Rapid Glo’** (≤ 2 weeks) | **BiCAR-like**  (1-2 weeks) | **Rapid Glo**  (≤ 1 week) | **Standard dosing** (Pivotal trial^) | **Standard dosing** (RWA^#^) |
| --- | --- | --- | --- | --- | --- |
| **CRS treatment** Steroids Tocilizumab Steroids + Toci  Other | 20 (24%) 4 (5%) 10 (12%) 6 (7%) 0 | 18 (28%) 4 (6%) 9 (14%) 5 (8%) 0 | 2 (10%) 0 1 (5%) 1 (5%)  0 | 58 (37%) 11 (7%) 15 (10%) 16 (10%) 16 (10%) | 24 (34%) 1 (4%) na 11 (39%) 12 (43%) |
| **ICANS treatment** Steroids Tocilizumab Other | 2 (2%) 1 (1%) 0 1 (1%) | 1 (2%) 0 0 1 (2%) | 1 (5%) 1 (5%) 0 0 | na na na na | na 2 (3%) 1 (1%)  1 (1%) |

CRS: Cytokine Release Syndrome; ICANS: Immune Effector Cell-associated Neurotoxicity Syndrome; Toci: Tocilizumab. ^ Registration trial, *Dickinson et al. NEJM 2022. # Shumilov, Wurm-Kuczera, Kerkhoff et al. Blood Advances 2024;*

**Supplemental Table 4: Grade ≥ 3 infections with accelerated Glo**

| **Infection grade ≥ 3 (n=8)** | **Accelerated Glo  (‘BiCAR-like’ + ’Rapid Glo’)** (≤ 2 weeks) | **BiCAR-like**  (1-2 weeks) | **Rapid Glo** (≤ 1 week) |
| --- | --- | --- | --- |
| **Grade 3** Gramnegative bacteria Cholecystitis Urinary tract infection Port infection CMV reactivation Candida albicans Unknown origin | 1 (1%) 1 (1%) 1 (1%) 1 (1%) 1 (1%) 1 (1%) 1 (1%) | 1 (2%) 1 (2%) 0 1 (2%) 1 (2%) 1 (2%) 1 (2%) | 0 0 1 (5%) 0 0 0 0 |
| **Grade 4** Aspiration pneumonia | 1 (1%) | 1 (2%) | 0 |
| **Grade 5** | 0 | 0 | 0 |

CMV: Cytomegalovirus

**Supplemental Table 5: ICU admissions**

| **ICU admissions (n=6)** | **Accelerated Glo  (‘BiCAR-like’ + ’Rapid Glo’)** (≤ 2 weeks) | **BiCAR-like**  (1-2 weeks) | **Rapid Glo** (≤ 1 week) |
| --- | --- | --- | --- |
| CRS monitoring ICANS Grade 2 Aspiration pneumonia  Tracheal obstruction Cholecystitis Tumor lysis grade 4 and AKI | 1 (1%) 1 (1%) 1 (1%) 1 (1%) 1 (1%) 1 (1%) | 1 (2%) 0 1 (2%) 1 (2%) 1 (2%) 1 (2%) | 0 1 (5%) 0 0 0 0 |

**Supplemental Table 6: Response parameters**

| **Response  Parameter** | **Accelerated Glo (‘BiCAR-like’+ ’Rapid Glo’** (≤ 2 weeks) | **BiCAR-like**  (1-2 weeks) | **Rapid Glo** (≤ 1 week) | **Standard dosing** (Pivotal trial^) | **Standard dosing**  (RWA^#^) |
| --- | --- | --- | --- | --- | --- |
| **No. of patients with staging** | 71 (86%) | 56 (88%) | 15 (79%) | 155 | 70 |
| **Median PFS** | 6.3 months | 9.9 months | 3.4 months | 4.9 months | 3.6 months |
| **Median OS** | 21.5 months | 21.5 months | NR | 11.5 months | 5.7 months |
| **Best response:** ORR CR or CMR PR SD or MR PD PR 🡪 CR | 44 (53%) 10 (12%) 34 (41%) 9 (11%) 18 (22%) 2 (2%) | 35 (55%) 10 (16%) 25 (39%) 8 (13%) 13 (20%) 2 (3%) | 9 (47%) 0 9 (47%) 1 (5%) 5 (26%) 0 | 52% 9% 13% na na na | 32 (46%) 29 (27%) 13 (19%) 1 (5%) 31 (44%) 7 (10%) |
| **Median time to response** | 1.2 months | 1.4 months | 1.1 months | TT CR:  1.4 months | 1.8 months |
| **Median duration of response** | 2.8 months | 2.9 months | 2.3 months | 14.4 months | 3.2 months |
| **Median time to follow up** | 5.1 months | 6.1 months | 4.2 months | 12 months | 5 months |
| **Status at last contact** Alive Dead | 66 (80%) 17 (20%) | 48 (75%) 16 (25%) | 18 (95%) 1 (5%) | na na | 27 (39%) 43 (61%) |

ORR: overall response rate; CR: complete remission; PR: partial remission; SD: stable disease; PD: progressive disease; NR: not reached; TT-CR: time to complete response. PFS: progression-free survival; OS: overall survival. Comparisons to external cohorts are descriptive and not based on matched populations. ^*Dickinson et al. NEJM 2022. # Shumilov, Wurm-Kuczera, Kerkhoff et al. Blood Advances 2024.*

**Supplemental Table 7: CRS rates stratified by treatment modality (monotherapy vs combination therapy)**

| **Parameter** | **All patients with accelerated dosing** | **Monotherapy** | **Combination Therapy** | **Significance  testing** |
| --- | --- | --- | --- | --- |
| **No. of patients** | 83 | 72 | 11 | - |
| **CRS** None Grade 1 Grade 2 | 39 (47%) 44 (53%) 30 (36%) 9 (11%) | 32 (44%) 40 (56%) 23 (32%) 9 (13%) | 7 (64%) 4 (36%) 7 (64%) 0 | *P* = 0.33 (Fisher’s exact test) |
